# White matter microstructural degeneration is spatiotemporally correlated with tau accumulation in Autosomal Dominant Alzheimer disease

**DOI:** 10.1002/alz70862_109793

**Published:** 2025-12-23

**Authors:** Nicole S. McKay, Stephanie Doering, David A. Hoagey, Sarah J. Keefe, Jeremy F. Strain, Peter R Millar, Jason J. Hassenstab, Chengjie Xiong, Eric McDade, Randall J. Bateman, Brian A. Gordon, Tammie L.S. Benzinger

**Affiliations:** ^1^ Washington University in St. Louis, School of Medicine, St. Louis, MO USA; ^2^ Washington University in St. Louis, St. Louis, MO USA; ^3^ Department of Neurology, Washington University School of Medicine, St. Louis, MO USA; ^4^ Washington University in St. Louis School of Medicine, St. Louis, MO USA

## Abstract

**Background:**

While amyloid correlates with white matter hyperintensities, tau distinctly impacts white matter microstructure through axonal injury from its prion‐like spread. Tau accumulation and white matter microstructural decline begin prior to clinical symptom onset, emphasizing their pivotal role in AD manifestation. Autosomal dominant AD (ADAD) has a relatively uniform phenotype that can be leveraged to investigate the preclinical relationship between tau accumulation and white matter microstructural health. Those with ADAD exhibit early pathology with few age‐related comorbidities, and the high penetrance of ADAD mutations enables precise disease staging relative to expected symptom onset.

**Method:**

Using data from the Dominantly Inherited Alzheimer Network, we characterized white matter integrity in ADAD mutation‐carriers and non‐carrier siblings using fractional anisotropy, mean diffusivity, axial diffusivity, and radial diffusivity. Tract‐based spatial statistics summarized these metrics across the brain, and we evaluated the emergence of abnormality relative to disease stage. Probabilistic tractography was conducted and summary white matter indices were extracted from the cingulum bundle and uncinate fasciculus, tracts connected to hubs of tau accumulation.

**Result:**

Whole‐brain analyses indicated that symptomatic mutation‐carriers have significantly different white matter microstructure compared to asymptomatic mutation‐carriers and non‐carriers. In mutation‐carriers, these abnormalities emerged concurrently with progression in symptomatic status. In contrast, our tract‐of‐interest analyses observed differences in white matter microstructure for all mutation‐carriers compared to non‐carriers. These changes within the cingulum bundle and uncinate fasciculus were determined to emerge five years prior to expected year of symptom onset.

**Conclusion:**

While global white matter differences are detected between mutation‐carriers and non‐carriers close to symptom onset, tracts that are adjacent to regions of significant tau accumulation show microstructural abnormality much earlier. This confirms that ADAD white matter decline does not occur uniformly across the brain, and may suggest a spatial correlation with tau. The timing of tract‐specific decline may also support that white matter damage occurs downstream of tau accumulation and spread. Given that white matter is critically important for transduction and integration of signals across disparate brain regions, the continued characterization of tau and white matter progression may provide important insight into the mechanism through which tau drives cognitive symptom onset in ADAD.

